# CDH6‐activated αIIbβ3 crosstalks with α2β1 to trigger cellular adhesion and invasion in metastatic ovarian and renal cancers

**DOI:** 10.1002/1878-0261.12947

**Published:** 2021-05-02

**Authors:** Rubén A. Bartolomé, Javier Robles, Ángela Martin‐Regalado, Laura Pintado‐Berninches, Miranda Burdiel, Marta Jaén, Carmen Aizpurúa, Juan I. Imbaud, José Ignacio Casal

**Affiliations:** ^1^ Department of Biomolecular Medicine Centro de Investigaciones Biológicas CSIC Madrid Spain; ^2^ Protein Alternatives SL Tres Cantos Spain

**Keywords:** CDH6, αIIbβ3 integrin, α2β1 integrin, ovarian and renal cancer, metastasis, platelets

## Abstract

Cadherin 6 (CDH6) is significantly overexpressed in advanced ovarian and renal cancers. However, the role of CDH6 in cancer metastasis is largely unclear. Here, we investigated the impact of CDH6 expression on integrin‐mediated metastatic progression. CDH6 preferentially bound to αIIbβ3 integrin, a platelet receptor scarcely expressed in cancer cells, and this interaction was mediated through the cadherin Arginine–glycine–aspartic acid (RGD) motif. Furthermore, CDH6 and CDH17 were found to interact with α2β1 in αIIbβ3^low^ cells. Transient silencing of *CDH6*, *ITGA2B,* or *ITGB3* genes caused a significant loss of proliferation, adhesion, invasion, and lung colonization through the downregulation of SRC, FAK, AKT, and ERK signaling. In ovarian and renal cancer cells, integrin αIIbβ3 activation appears to be a prerequisite for proper α2β1 activation. Interaction of αIIbβ3 with CDH6, and subsequent αIIbβ3 activation, promoted activation of α2β1 and cell adhesion in ovarian and renal cancer cells. Additionally, monoclonal antibodies specific to the cadherin RGD motif and clinically approved αIIbβ3 inhibitors could block pro‐metastatic activity in ovarian and renal tumors. In summary, the interaction between CDH6 and αIIbβ3 regulates α2β1‐mediated adhesion and invasion of ovarian and renal cancer metastatic cells and constitutes a therapeutic target of broad potential for treating metastatic progression.

## Graphical abstract

Integrin activation plays a key role in cancer metastasis. However, a potential cooperative effect between different integrins was previously unknown in solid tumors. Here, we demonstrated the role of CDH6 in regulating metastatic progression of ovarian and renal cancer by promoting the crosstalk between αIIbβ3 and α2β1 integrins. Based on these findings, our study unveils new strategies for the treatment of ovarian and renal cancer metastasis.

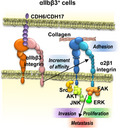

**Abbreviations**
BCECE‐AM: 2',7'‐bis‐(2‐carboxyethyl)‐5‐(y‐6)‐carboxyfluorescein, acetoxymethyl ester
CDH17: Cadherin 17
CDH6: Cadherin 6
ITGA2B: Integrin‐αIIb gene
ITGB3: Integrin‐β3 gene
PAX8: Paired‐box gene 8
RGD: Arginine–glycine–aspartic acid

## 1 Introduction

Ovarian cancer is considered the fifth cause of cancer death in women [1] and roughly comprises the histological subtypes of serous (75%), endometrioid (10%), clear cell (10%), and mucinous (3%), with distinct molecular alterations [2]. In ovarian carcinoma, 70% of patients initially present with disseminated disease, which decreases the survival rate to 17% [3]. Ovarian cancer causes a rapid and early metastasis to peritoneum and omentum, associated with ascites formation [4]. Further dissemination sites for metastatic ovarian cancer include liver and lung metastasis [5, 6]. Molecularly, the Arginine–glycine–aspartic acid (RGD)‐containing cadherin 6 (CDH6, K‐cadherin) is often highly expressed and has been determined in 70% of high‐grade serous, 45% of low‐grade serous, 27% of clear cell, 25% of endometrioid, and 3.6% of mucinous cases, with a preferential expression in stage III cancers, except for high‐grade serous, which show invariably high expression [7]. Cadherin 17 (CDH17, LI‐cadherin), another RGD cadherin [8], appears to be highly expressed in mucinous ovarian cancers, according to the Human Protein Atlas database (www.proteinatlas.org/ENSG00000079112‐CDH17/pathology/ovarian+cancer#ihc) [9], but has yet to be further characterized in ovarian cancer.

Renal cancer is one of the ten most common tumors, with 403 000 new cases diagnosed worldwide and 175 000 deaths in 2018 [10]. Clear‐cell renal carcinoma is the most common subtype (75%), followed by papillary carcinomas (16%). CDH6 is also highly expressed in renal cancers (in 47% of clear cell and 66 % of papillary subtypes). The survival rate in renal cancer is very good (92%) when the tumor is confined to the kidney, but drops to 12% after metastasis. The main organs for metastasis are lung, bone, liver, and brain [11]. Overall, CDH6 overexpression is observed in tumors originating from developmentally related Mullerian lineages (e.g., ovarian, renal, and thyroid) [12]. The transcription factor Paired‐box gene 8 (PAX8) is common to these three lineages and regulates CDH6 expression [13, 14]. PAX8 is also highly expressed in 90% of metastatic tumors of Mullerian origin [15]. In adults, CDH6 expression is restricted to the proximal renal tubules and platelets [16]. Two splicing variants have been reported for CDH6 [17]. The canonical isoform 1 contains all the type II cadherin domains. Isoform 2 (the ‘short’ form) of CDH6 contains a very short cytoplasmic domain that has lost the conserved catenin‐binding sites and mimics the very short cytoplasmic domain of CDH17.

Cadherins, together with integrins, play a major role in metastatic progression and organ colonization [8, 18, 19]. Cadherin studies in ovarian cancer have been mostly restricted to E‐cadherin and N‐cadherin due to their involvement in the epithelial–mesenchymal transition [20]. Of note, however, E‐cadherin expression and N‐cadherin expression fail to correlate with disease progression in renal carcinoma [21, 22]. In contrast, CDH6 levels have been associated with renal carcinomas with poor prognosis [16] and metastasis [23]. Previously, we described the capacity of CDH17 and CDH5 (VE‐cadherin) to promote liver and lung metastasis, by interacting with α2β1 integrin via their internal RGD motif, in different tumors (colorectal, melanoma, and breast cancer) [24, 25, 26]. In accordance with these findings, CDH17 RGD‐specific monoclonal antibodies (mAbs) inhibited the metastatic colonization in colorectal cancer and melanoma animal models [27]. We hypothesized that, in a similar way, CDH6 might bind and activate α2β1 integrin in ovarian and renal cancer cells, being an additional target for those mAbs.

CDH6 contains a phylogenetically well‐preserved RGD motif [28], which plays a critical role in platelet aggregation and blood coagulation mediated by the binding of CDH6 to αIIbβ3 integrin [29]. Interestingly, CDH6 contains the only cadherin RGD motif conserved not only in human and mouse but also in almost every sequenced vertebrate specie, underscoring the critical function of this RGD motif [28]. Integrin αIIbβ3 expression has not been described in cancer cells, except a few reports in melanoma and prostate cancer metastasis [30, 31]. As there is no information about αIIbβ3 expression in ovarian or renal cancer, no functional link between CDH6 and αIIbβ3 has been previously investigated. According to the described interaction in platelets, we decided to investigate whether a similar association occurs in cancer cells.

We have now investigated the expression levels and different molecular interactions of CDH6, CDH17, αIIbβ3, and α2β1 integrins in ovarian and renal cancer. Furthermore, we have characterized the molecular mechanisms underpinning CDH6‐mediated integrin crosstalk activation in metastatic progression. Notably, our results suggest that blocking the CDH6–integrin interaction with either RGD‐specific mAbs or αIIbβ3 integrin inhibitors represents a promising therapeutic strategy for treating ovarian and renal cancer metastasis.

## 2 Materials and methods

### 2.1 Cell lines, antibodies, peptides, inhibitors, and siRNAs

Ovarian OVCAR3 cells were purchased from ATCC (Manassas, VA, USA), SKOV‐3 cells were obtained from Sigma‐Aldrich (St. Louis, MO, USA), and A2780 cells were a kind gift from J.F. Diaz (CIB‐CSIC). Renal cancer cells (RCC4, 786‐O) were provided by M.J. Calzada (Hospital de la Princesa, Madrid) and CAKI1 by P. Real (CNIO). Colon cancer HT‐29 cells were purchased from ATCC. All cell lines were cultured in RPMI 1640 containing 10% FBS (Invitrogen, Carlsbad, CA, USA) and antibiotics at 37 °C in a 5% CO_2_ humidified atmosphere, except for OVCAR3, which was cultured in RPMI 1640 with 20% FBS and insulin (0.01 mg·mL^−1^) (Sigma‐Aldrich).

Human CDH6 ORFeome V8.1 (Broad) clone CCSBo5058E0381109D encoding for a complete transcript was obtained from Source Bioscience (Nottingham, UK). Subsequent in‐house sequencing of this clone showed that corresponds to isoform 2 of CDH6, which is missing a large portion of the cytoplasmic domain. siRNAs against human CDH6 (#1 SASI_Hs01_00067020, #2 SASI‐Hs02_00338600), β3 integrin subunit (#1 SASI_Hs01_00174219, #2 SASI_Hs01_00174221), αIIb integrin subunit (#1 SASI_Hs01_0075094, #2 SASI_Hs01_0075095), CDH17 [24], or control (AUUGUAUGCGAUCGCAGACdTdT) were purchased from Sigma‐Aldrich and transfected using Jet Prime reagent (Polyplus, Illkirch, France) according to manufacturer's instructions. CDH17 (VSLRGDTRG) and CDH6‐derived peptides (DQDRGDGSL) were synthesized as described [26]. Eptifibatide was purchased from Selleckchem (Houston, TX, USA).

Purified RGD‐cadherin‐specific monoclonal antibodies 6.6.1 and 25.4.1 were prepared by ProAlt SL (Tres Cantos, Spain) [27]. Antibodies for CDH6 (K‐13; for immunoprecipitation), CDH17 (H‐167), integrin subunits α2 (P1E6), β1 (K‐20), αV (H‐2), and β3 (B‐7), FAK (D‐1), and RhoGDI (G‐2) were purchased from Santa Cruz Biotechnology (Dallas, TX, USA). Antibodies for phospho‐AKT, phospho‐extracellular signal‐regulated kinase, phospho‐c‐Jun N‐terminal kinase, (#4060, #9101, #9255, respectively) and their total counterparts (#9272, #4695, #9258), as well as phospho‐SRC (#2101) were from Cell Signaling Technology (Danvers, MA, USA). CDH6 antibody (M06353) for immunofluorescence was from Boster (Pleasanton, CA, USA), whereas antibodies for Src (AF3389) and CDH6 (AF2715) for Western blot and flow cytometry were from R&D Systems (Minneapolis, MN, USA). β‐Tubulin antibody (ab21057) and phospho‐focal adhesion kinase (611722) were from Abcam (Cambridge, UK) and BD Transduction Laboratories (Franklin Lakes, NJ, USA), respectively. β1 and β3 integrin‐specific antibodies for high‐affinity conformation were from BD Biosciences, San Diego, CA, USA (HUTS21) and Merck, Kenilworth, NJ, USA (MABT27), respectively. Anti‐αIIb integrin (clone 2BC1) was a gift from C. González‐Manchón (CIB).

### 2.2 Western blot

Cells were detached with 2 mm EDTA in PBS, washed, and then lysed with protease (Roche, Basel, Switzerland) and phosphatase inhibitors (Sigma‐Aldrich) in GST‐FISH lysis buffer (1% Igepal, 100 mm NaCl, 2 mm MgCl_2_, 10% glycerol, 50 mm Tris/HCl). Protein extracts were resolved in 7.5% SDS/PAGE and transferred to nitrocellulose membranes, which were incubated with 5% skimmed milk in PBS, followed by incubation with primary antibodies and, finally, with HRP‐coupled secondary antibodies (Thermo‐Scientific, Waltham, MA, USA). Bands were visualized using West Pico Chemiluminescent Substrate (Thermo‐Scientific) and quantified using quantity one software (Bio‐Rad, Hercules, CA, USA).

### 2.3 Flow cytometry

For flow cytometry, 1 × 10^5^ cells were detached with 2 mm EDTA in PBS, washed with PBS, resuspended in PBS with gamma‐globulin (20 µg·mL^−1^), incubated at 4 °C with primary antibodies (10 µg·mL^−1^) for 30 min, washed again, and finally incubated with Alexa Fluor 488‐labeled secondary antibodies (anti‐mouse IgG or anti‐rabbit IgG; Dako, Glostrup, Denmark). Fluorescence was quantified in a Coulter Epics XL cell cytometer. Mean fluorescence intensities are indicated for each antibody.

### 2.4 Analysis of β1 and β3 integrin activation

Integrin activation assays were carried out as previously described [27]. Cells were detached with 2 mm EDTA in PBS, washed, resuspended in Dulbecco's Modified Eagle's medium (DMEM), and incubated with 1 µg·mL^−1^ CDH17 or CDH6‐derived 9‐amino acid RGD peptides, antibodies (10 µg·mL^−1^) or 10 nm eptifibatide for 40 min at 37 °C. After incubation, cells were subjected to flow cytometry assays as above using specific antibodies for high‐affinity conformation of β1 and β3 integrin. The activation status of the integrins was referenced against the fluorescence intensity of nontreated cells.

### 2.5 Confocal microscopy

Protein localization was carried out by fluorescence microscopy. For this purpose, cells were fixed in 4% formaldehyde solution (Sigma‐Aldrich) at room temperature for 10 min. Cells were washed with PBS, permeabilized with 0.2% Triton X‐100, and blocked with 2% BSA (Sigma‐Aldrich) and 10% goat serum before incubation with anti‐CDH6, anti‐α2, and anti‐αIIb integrin subunit antibodies at room temperature for 1 h. Finally, after washing with PBS, cells were incubated with secondary antibodies labeled with Alexa Fluor 488 or Alexa Fluor 647 for 1 h. Images were acquired with a Confocal Spectral Leica TCS SP5 using a HCX PL APO CS 63×/1.4‐0.60 oil. Image processing was performed using imagej (National Institutes of Health, Bethesda, MD, USA) and LAS‐AF 1.8.1 leica software (Wetzlar, Germany). Colocalization was quantified using Pearson´s correlation included in the JACoP software package [32].

### 2.6 Immunoprecipitation

Cells were lysed with GST‐FISH buffer, and 500 µg of cell lysates was incubated with 5 µg of anti‐CDH6, anti‐CDH17, anti‐αIIb integrin, or control antibodies. Immunoprecipitation was performed as previously described procedures [26].

### 2.7 Proliferation assays

Proliferation assays were carried out as previously described [26, 27].

### 2.8 Cell adhesion and invasion assays

For adhesion assays, cancer cells were starved for 3 h, detached, labeled with BCECF‐AM (Sigma‐Aldrich) for 45 min, and incubated in serum‐free DMEM in the presence of the indicated antibodies (10 µg·mL^−1^) or eptifibatide (10 nm) for 10 min. Then, 6 × 10^4^ cells were loaded in 96‐well plates previously coated with Matrigel (0.4 µg·mm^−2^; BD Biosciences), blocked with 0.5% BSA (Sigma‐Aldrich), and incubated for 25 min at 37 °C. Nonadhered cells were then removed by three washes with DMEM. Bound cells were lysed with 1% SDS in PBS, and the extent of the adhesion was quantified using the fluorescence analyzer POLARstar Galaxy (BMG Lab Technologies, Ortenberg, Germany).

Matrigel invasion assays were performed as previously described [27], in the presence of the indicated antibodies or eptifibatide (at the indicated concentrations).

### 2.9 Cell migration assay

Cells were seeded in 24‐well plates that had been previously coated covered with Matrigel (0.5 µL·mL^−1^). When cells reached confluence, a longitudinal incision was made in each well. Then, cells were incubated in serum‐free DMEM with the indicated antibodies (10 µg·mL^−1^) or eptifibatide (10 nm). Pictures were taken just after the incision and 24 h later. Migratory speed was calculated as the advanced distance of each flank in the 24‐h period.

### 2.10 RT‐PCR analysis

Cells were lysed in Tri Reagent (Sigma‐Aldrich), and RNA was extracted and reverse‐transcribed using Moloney murine leukemia virus retrotranscriptase (Promega, Madison, WI, USA). PCR used TaqDNA polymerase (Invitrogen Corp.) and the following primers: 5′‐TGCCTGTGGTCATTTCAGAC‐3′ (forward), 5′‐GCCTCATAGGCGTAAGTGG‐3′ (reverse for isoform 1), 5′‐AAAGGGCCCTCATCATACAC‐3’ (reverse for isoform 2) for CDH6; 5′‐CGGCCATCACGCCACAGTTTC‐3′ (forward), 5′‐GGCTGAGAACGGGAAGCTTGT‐3′ (reverse) for GAPDH; and 5′‐CATGTACGTAGCCATCCAGGC‐3′ (forward), and 5′‐CTCTTTGATGTCACGCACGAT‐3′ (reverse) for murine β‐actin. The PCR program was 35 cycles of 30‐s denaturation at 94 °C, 30‐s annealing at 57 °C, and 45‐s polymerization at 72 °C.

### 2.11 *In vivo* animal experiments

The Ethical Committee of the CSIC and Comunidad de Madrid approved all the protocols (PROEX 252/15) used in these experiments. Swiss nude mice (Crl:NU(Ico)‐*Foxn1^nu^*) were bred and maintained in the Animal Facility of CIB‐CSIC under standard conditions. Swiss nude mice 8‐ to 10‐week‐old were inoculated intravenously or in‐spleen with 1 × 10^6^ SKOV‐3 or 786‐O cells. Then, mice were euthanized 72 h after inoculation, and RNA was isolated using Tri Reagent from lungs and liver. RNA was analyzed by RT‐PCR to amplify human GAPDH and, as loading control, murine β‐actin.

### 2.12 *In silico* studies

For *in silico* analyses, independent external cohorts of patients were used: for ovarian cancer, public databases GSE2613 (with 107 tumor samples) and GSE26712 (with 195 tumor samples and 10 healthy ovarian tissue samples); for renal cancer, TCGA Renal 2 (with 1071 tumor samples and 441 healthy renal tissue samples); and for clear‐cell renal cancer, GSE22541 (with 44 metastasis samples and 24 primary tumor samples). The values of mRNA expression levels were normalized by calculating the z‐scores prior to statistical analyses. Populations with high or low expression were divided by the best cutoff method. The GEPIA web tool (http://gepia.cancer‐pku.cn/) and Oncomine platform (https://www.oncomine.org) were used to analyze the TCGA Renal 2 database in prognostic studies and gene expression, respectively.

### 2.13 Statistical analysis

Data were analyzed by Student's *t*‐test (for two conditions) or one‐way ANOVA (for more than two conditions), followed by Tukey–Kramer multiple comparison test. Prognostic studies of patient survival were analyzed by log‐rank test. For all analyses, the minimum acceptable level of significance was *P* < 0.05.

## 3 Results

### 3.1 Expression of CDH6, CDH17, α2β1, and αIIbβ3 integrins in ovarian and renal cancer cells

**Fig. 1:**
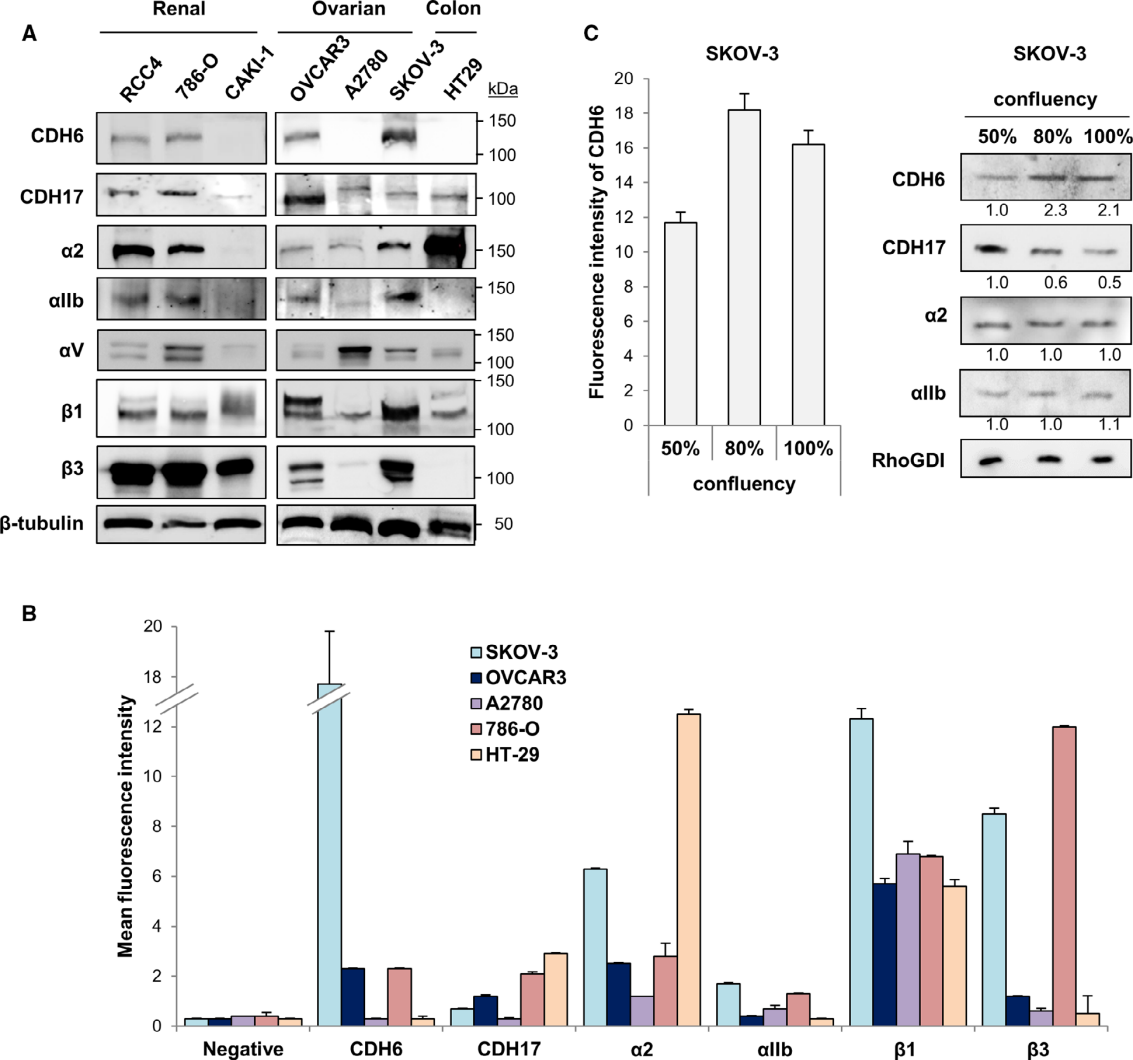
Expression of RGD Cadherins and integrins in renal and ovarian cancer cells. (A) Western blot analysis of the expression of CDH6, CDH17, and the indicated integrin subunits in renal cancer, ovarian cancer, and colorectal cancer cells. β‐Tubulin expression was used as loading control. (B) Flow cytometry analysis of cadherin and integrin expression in the indicated cell lines. (C) Changes in the expression of the indicated cadherins and integrin subunits as a function of SKOV‐3 cell confluency by flow cytometry (left) and Western blot analysis (right). All results are representative of at least three independent experiments; error bars indicate standard deviation.

We first investigated total and plasma membrane expression of CDH6 and CDH17, together with α2β1 and αIIbβ3 integrins, in different ovarian and renal cancer cell lines, using the HT29 colorectal cancer cell line as positive control for expression of CDH17 and α2β1 integrin. For total protein levels, CDH6 was highly expressed in ovarian (OVCAR3 and SKOV‐3) and renal (RCC4 and 786‐O) cancer cell lines except in CAKI‐1 and A‐2780, while CDH17 was expressed in all cell lines, with RCC4, 786‐O, and OVCAR3 exhibiting the highest amounts (Fig. 1A). Notably, all cell lines were positive for α2, αIIb, β1, and β3 integrin subunits, except for CAKI‐1 cells being negative for α2 and αIIb (Fig. 1A). αv‐integrin subunit was expressed in all cell lines, with CAKI‐1 and OVCAR3 showing the lowest expression. Using flow cytometry to investigate the surface accessibility, a higher expression of CDH6 was observed in the plasma membrane of SKOV‐3 with respect to OVCAR3 and 786‐O, while A‐2780 cells were negative (Fig. 1B). CDH17 was detected in all cell lines except A2780. The αIIb and β3 subunits were mainly observed in the membrane of SKOV‐3 and 786‐O cells, with OVCAR3 expressing very low levels (Fig. 1B). Overall, ovarian and renal cancer cell lines exhibited co‐expression of α2β1 and αIIbβ3 integrins, with higher expression of α2β1 integrin at the plasma membrane. Next, we investigated the effect of cellular confluence on the expression of cadherins and integrins (Fig. 1C). A clear increase of CDH6 expression was observed at high cellular confluence in SKOV‐3 cells, consistent with its role in the strengthening of cell–cell adhesions. In contrast, no increase or a minor decrease was observed for α2 and αIIb subunits and CDH17, respectively.

To assess the clinical relevance of CDH6 and αIIbβ3 integrin, we examined their expression and prognostic value in ovarian and renal cancer. Overall, CDH6 expression was higher in renal cancer samples than in normal kidney tissue (Fig. S1A). However, only the papillary renal cancer subtype showed an increase of αIIbβ3 integrin expression levels (Fig. S1A). Furthermore, CDH6 and αIIbβ3 integrin expression were increased in the metastatic stage of clear‐cell renal carcinoma (Fig. S1B). In ovarian cancer, there was a significant overexpression of CDH6 as compared to ovarian tissues, but αIIbβ3 expression remained constant (Fig. S1C). In addition to the association between CDH6 and a worse prognosis in ovarian serous carcinoma, αIIbβ3 associated with poor prognosis in clear‐cell and papillary renal carcinomas as well as in ovarian cancer (Fig. S2). Together, these results supported further investigation of CDH6 and αIIbβ3 integrin in ovarian and renal cancer.

### 3.2 CDH6 preferentially associates with αIIbβ3 integrin in cancer cells

**Fig. 2:**
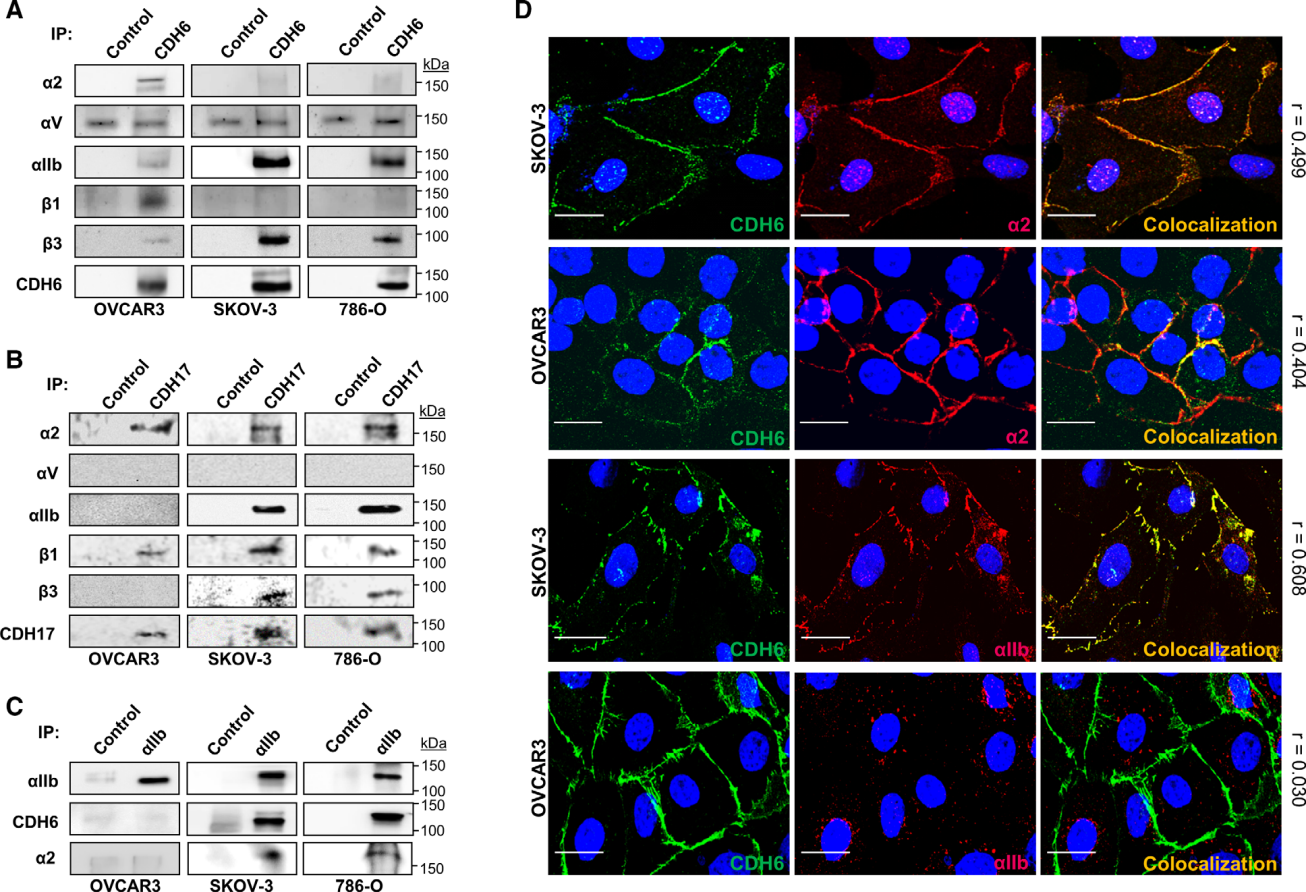
CDH6 binds αIIbβ3 or α2β1 integrins in different cell lines. (A, B, C) The indicated cells were immunoprecipitated using anti‐CDH6, anti‐CDH17, anti‐αIIb, or control antibodies. Immunoprecipitates were analyzed by Western blot using the indicated antibodies. (D) SKOV‐3 and OVCAR3 cells were used to determine CDH6, α2, and αIIb integrin subunits colocalization by confocal microscopy. Pearson’s correlation coefficient was calculated for each colocalization experiment (right). Bar size: 20 µm.

Next, we investigated the association of CDH6 and CDH17 with α2β1 or αIIbβ3 integrin in SKOV‐3, OVCAR3, and 786‐O cells using immunoprecipitation (IP) and Western blot. By IP, we found a preferential association of CDH6 with αIIbβ3 in SKOV‐3 and 786‐O cells and with α2β1 in OVCAR3 (Fig. 2A). Regarding CDH17 IP, we observed an association with α2β1 in OVCAR3 and with both integrins in SKOV‐3 and 786‐O (Fig. 2B). Consistently, αIIb IP retrieved a high amount of CDH6 in SKOV‐3 and 786‐O cells, but not in OVCAR3 cells (Fig. 2C). To note that the α2 subunit was also present in the αIIb IP, suggesting some interaction between integrins. As a confirmation, confocal microscopy showed the colocalization of CDH6 and the α2 subunit in the plasma membrane of SKOV‐3 and OVCAR3, and CDH6 and αIIb in SKOV‐3 (Fig. 2D). In contrast, no colocalization of CDH6 and αIIb was observed in OVCAR3. Determination of Pearson´s correlation coefficient values (Fig. 2D) supports the observed colocalizations. Collectively, these data indicate the capacity of CDH6 to interact preferentially with the αIIbβ3 integrin, whereas its association with α2β1 was restricted to cells lacking αIIbβ3 in the plasma membrane.

### 3.3 The CDH6–αIIbβ3 integrin interaction regulates the pro‐invasive properties of ovarian and renal cancer cells through its RGD motif

**Fig. 3:**
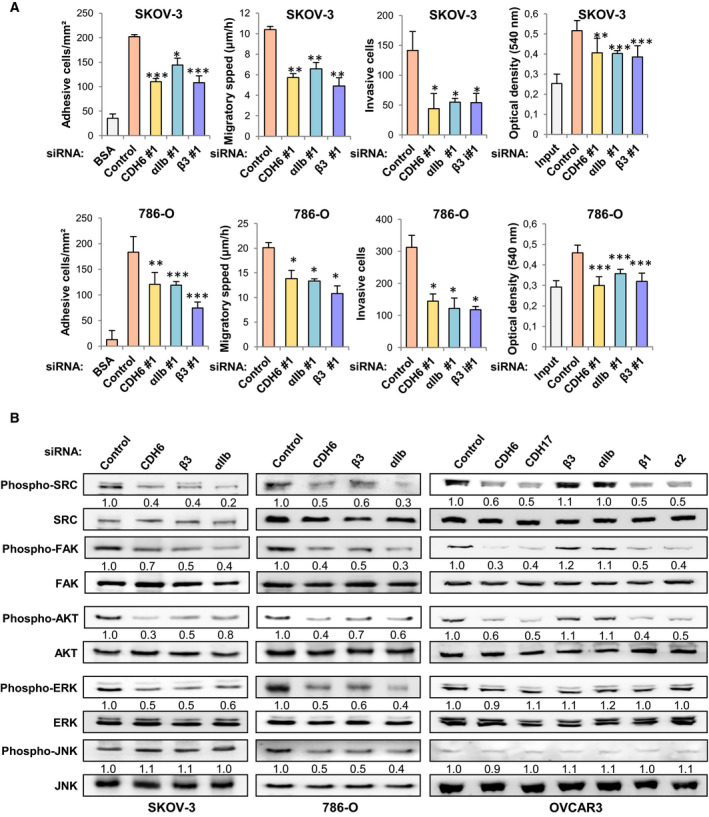
CDH6 and αIIbβ3 integrin regulate cell adhesion, migration, invasion, and proliferation in ovarian and renal cancer cells. (A) SKOV‐3 and 786‐O cells were transfected with siRNAs for the indicated genes and subjected to cell adhesion, wound healing, cell invasion, or MTT assays. Transient silencing of CDH6, αIIb, or β3 integrin subunits caused a significant decrease in cell adhesion/migratory speed/cell invasion/proliferation (*P < 0.05; **P < 0.01; ***P < 0.001), according to ANOVA tests. (B) The same transfectants were analyzed by Western blot to examine the phosphorylation status of the indicated signaling proteins. Blots were re‐probed with antibodies against the total signaling proteins as loading controls. All results are representative of at least three independent experiments; error bars indicate standard deviation.

To explore the impact of the CDH6–αIIbβ3 integrin interaction on the tumorigenic activity of SKOV‐3 and 786‐O cells, we knocked down CDH6, CDH17, αIIb, and β3 integrin subunits using transient silencing with two different siRNAs for each (Fig. S3A). A similar decrease in adhesion, migration, and, in particular, invasion, with a minor effect in cell proliferation, was observed after silencing of CDH6, αIIb, or β3, in both cell lines with both siRNAs. These results suggest a common functional mechanism (Figs 3A and Fig. S3B). Of note, knocking down CDH17 and CDH6 caused similar effects in reducing the pro‐metastatic properties of ovarian and renal cancer cells, likely through the direct interaction of CDH17 with α2β1 integrin (Fig. S3B). To confirm the role of the RGD motif in these tumorigenic effects, we expressed the shorter isoform 2 of CDH6 in CDH6‐negative A‐2780 cells (Fig. S4A, B). Isoform 2, containing the RGD motif and lacking the catenin‐binding domain, caused a substantial increase in cell adhesion, migration, invasion, and proliferation capacities with respect to the parental cells (Fig. S4C). These results confirmed that integrin activation and pro‐metastatic effects in ovarian cancer cells are RGD‐mediated.

Next, we investigated the effect of transient knocking down CDH6, αIIb, and β3 on the integrin signaling pathway in SKOV‐3 and 786‐O cells. Silencing any one of these three genes reduced the phosphorylation and activation of FAK, AKT, ERK, and SRC kinases in both cell lines, including JNK activation in 786‐O cells (Fig. 3B). It is noteworthy that signaling inhibition was observed despite the unaltered expression of the α2β1 integrin in these cells, suggesting that αIIbβ3 silencing inhibits the activation of α2β1. Previous integrin crosstalk between αIIbβ3 and α2β1 was reported in platelets, where either ligand binding to αIIbβ3 inhibits α2β1‐mediated binding to collagen or αIIbβ3 activation is a prerequisite for α2β1 activation [33, 34, 35].

### 3.4 Integrin crosstalk in ovarian and renal cancer cells

**Fig. 4:**
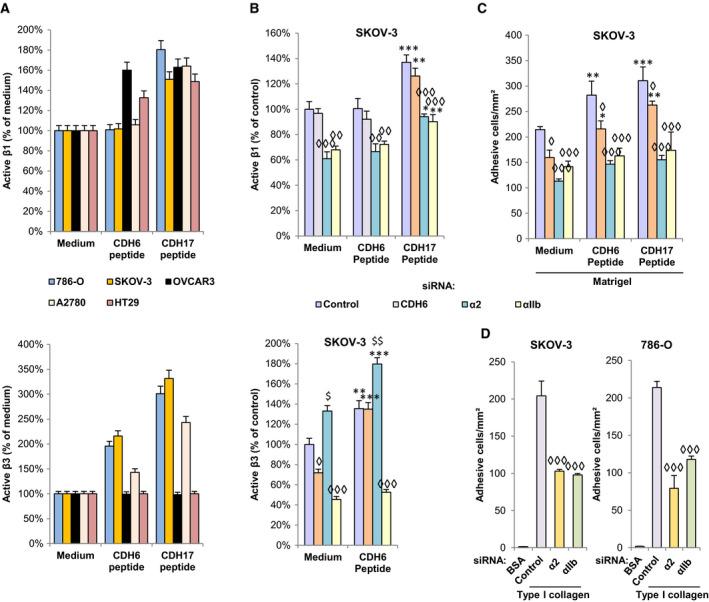
CDH6 and CDH17 promote the activation of β1 and β3 integrins. (A) Ovarian and renal cancer cell lines were exposed to RGD peptides of CDH6 or CDH17 and subjected to flow cytometry analyses to assess the activation status of β1 and β3 integrins. (B) SKOV‐3 cells were silenced for CDH6, α2, or αIIb integrin subunits, exposed to the RGD peptides and subjected to flow cytometry assays to detect β1 or β3 in high‐affinity conformation. (C) The same transfectants exposed to the indicated peptides were subjected to cell adhesion assays to Matrigel. (D) SKOV‐3 and 786‐O cells were silenced for α2 or αIIb integrin subunits and subjected to adhesion to collagen type I. Integrin activation or cell adhesion was significantly increased by the addition of RGD peptides (**P < 0.01; ***P < 0.001) or the silencing of α2 integrin subunit ($*P* < 0.05; $$*P* < 0.01) and significantly decreased by the silencing of CDH6, α2, or αIIb (◊*P* < 0.05; ◊◊◊*P* < 0.001), according to ANOVA tests. All results are representative of at least three independent experiments; error bars indicate standard deviation.

To further investigate the integrin crosstalk, we carried out different experiments using cadherin RGD peptides and gene silencing. First, we tested the impact of cadherin and integrin silencing in integrin activation in SKOV‐3 and 786‐O cells. CDH6 silencing inhibited β3, but not β1 activation, while CDH17 silencing inhibited β1 preferentially and β3 to a lesser extent (Fig. S5A). Furthermore, αIIb or α2 silencing abolished β1 activation, whereas only αIIb silencing inhibited β3 activation (Fig. S5B). Regarding RGD‐mediated activation, β1 was activated by the CDH17 peptide in all cell lines and by the CDH6 peptide in OVCAR3 (Fig. 4A). In contrast, both peptides activated β3 in the three cell lines that express this subunit in plasma membrane (Fig. 4A). We then investigated peptide activation combined with cadherin/integrin silencing in SKOV‐3. β1 activation was inhibited after silencing αIIb and α2 integrins, but not by CDH6 knockdown (Fig. 4B). Notably, β3 activation was inhibited after αIIb silencing but increased after α2 silencing (Fig. 4B). Next, we explored the role of both integrins in cell adhesion. Silencing of α2 or αIIb inhibited the adhesive capacity of the ovarian cancer cells in the presence of CDH6 and CDH17 peptides (Fig. 4C). Adhesion was specifically mediated by α2β1 integrin, as silencing of either integrin inhibited the adhesion to collagen type I, an exclusive ligand of α2β1 integrin (Fig. 4D). In summary, when both integrins are co‐expressed in the same cell line, the activation of αIIbβ3 is the necessary prerequisite for the activation of α2β1 integrin, which is the key integrin required for cell adhesion. In contrast, β3 activation is independent of α2β1.

### 3.5 Cadherin RGD‐specific monoclonal antibodies inhibit adhesion, migration, and invasion in ovarian and renal cancer cells

**Fig. 5:**
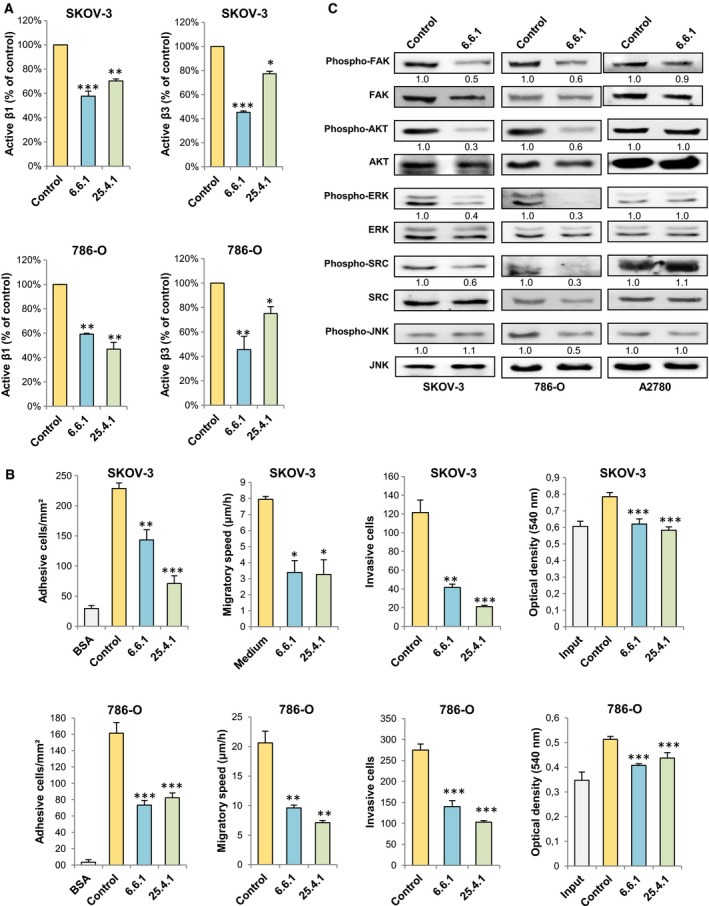
Anti‐RGD cadherin antibodies inhibit integrin activation, cell adhesion, migration, invasion, and proliferation in ovarian and renal cancer cells. (A) SKOV‐3 and 786‐O cells were exposed to the indicated anti‐RGD cadherin antibodies or control antibodies and analyzed in flow cytometry assays to assess the activation status of β1 and β3 integrins (A) or in cell adhesion, wound healing, cell invasion, or MTT assays (B). In (B), treatment with the anti‐RGD cadherin antibodies provoked a significant decrease in integrin activation/cell adhesion/migratory speed/cell invasion/proliferation (*P < 0.05; **P < 0.01; ***P < 0.001), according to ANOVA tests. (C) The indicated cell lines treated with the 6.6.1 antibody or a control antibody were analyzed by Western blot to assess the phosphorylation status of the indicated signaling proteins. Blots were re‐probed using antibodies against the total signaling proteins as loading controls. All results are representative of at least three independent experiments; error bars indicate standard deviation.

Previous studies underlined the inhibitory effect of CDH17 RGD‐specific mAbs in mouse models of liver and lung metastasis for colorectal cancer and melanoma, respectively [27]. Based on these results, we examined whether the cadherin RGD mAbs 6.6.1 and 25.4.1 were also effective in the blocking of ovarian and renal carcinomas progression. Both mAbs inhibited the activation of β1 and β3 in SKOV‐3 and 786‐O cells, although 6.6.1 was more effective for inhibiting β3 activation (Fig. 5A). Consequently, mAbs 6.6.1 and 25.4.1 strongly reduced the adhesion, migration, and invasion, and, to a lesser extent, proliferation, of SKOV‐3 and 786‐O cancer cells (Fig. 5B). For a further confirmation that these effects were integrin‐mediated, we explored the inhibitory effect of 6.6.1 on the integrin signaling pathway activation in the three cell lines. mAb 6.6.1 reduced the activation of Src, FAK, AKT, and ERK kinases in SKOV‐3 and 786‐O cells, without affecting JNK activation in SKOV‐3 (Fig. 5C). As a negative control, A‐2780 cells lacking CDH6 did not show alterations in the signaling pathways (Fig. 5C). Together, these data indicate the capacity of both mAbs to inhibit the pro‐metastatic activity in CDH6^high^ ovarian and renal cancer cells.

### 3.6 Eptifibatide inhibits ovarian and renal cancer cell progression

**Fig. 6:**
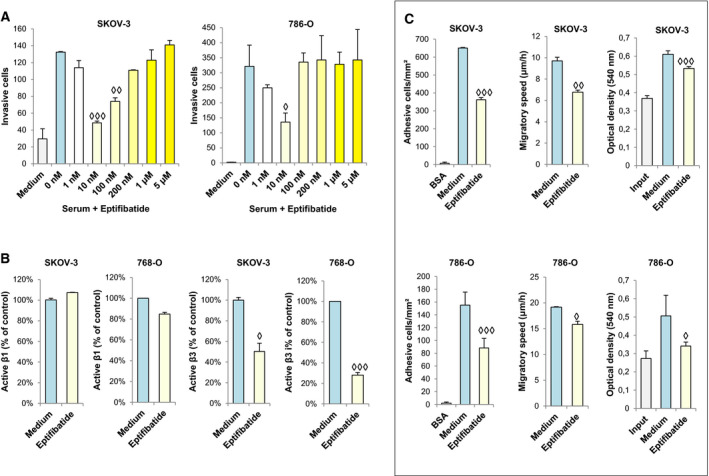
Eptifibatide blocks cell adhesion, migration, invasion, and proliferation in ovarian and renal cancer cells. (A) SKOV‐3 and 786‐O cells were analyzed in invasion assays through Matrigel in the presence of different concentrations of eptifibatide. (B,C) SKOV‐3 and 786‐O cells treated with eptifibatide (10 nm) were analyzed by (B) flow cytometry, to estimate the activation of β1 and β3 integrins, or (C) cell adhesion, wound healing, and MTT assays. Eptifibatide caused a significant decrease in integrin activation, cell adhesion, migratory speed, cell invasion, and proliferation (◊*P* < 0.05; ◊◊*P* < 0.01; ◊◊◊*P* < 0.001). All results are representative of at least three independent experiments. Error bars indicate standard deviation. Data were analyzed by Student’s t‐test (in assays of two conditions) and by ANOVA test (in assays of more than two conditions).

CDH6 binding to αIIbβ3 integrin induces platelet aggregation and thrombus formation [29]. Furthermore, αIIbβ3 integrin in metastasis has been associated with platelet/tumor cell interactions and tumor cell‐induced platelet aggregation [36]. Eptifibatide, a cyclic heptapeptide Lysine–glycine–aspartic acid mimetic, is a clinically used αIIbβ3 inhibitor that inhibits platelet aggregation by preventing the binding of RGD proteins (fibrinogen, von Willebrand factor) to αIIbβ3 integrin. We hypothesized a similar effect of eptifibatide on CDH6 binding. Therefore, we explored its capacity to block invasion capacity on Matrigel in SKOV‐3 and 786‐O cells. We found a dose‐dependent inhibition, with the highest antagonistic effect at 10 nm eptifibatide (Fig. 6A). In addition, we observed inhibition of the adhesion, migration, and proliferative capacities of SKOV‐3 cells at 10 nm eptifibatide (Fig. 6B). This antagonism of the metastatic capacities was shown to be specifically mediated through β3 by flow cytometry (Fig. 6C). The effect on cell adhesion confirms the indirect inhibition of α2β1 integrin [37]. Given the observed integrin crosstalk, the antimetastatic effect of eptifibatide might rely on an indirect inhibition of collagen‐binding by α2β1 integrin after blocking αIIbβ3 integrin activation.

### 3.7 CDH6 and αIIb and α2 integrins are required for lung but not liver homing in ovarian and renal cancer

**Fig. 7:**
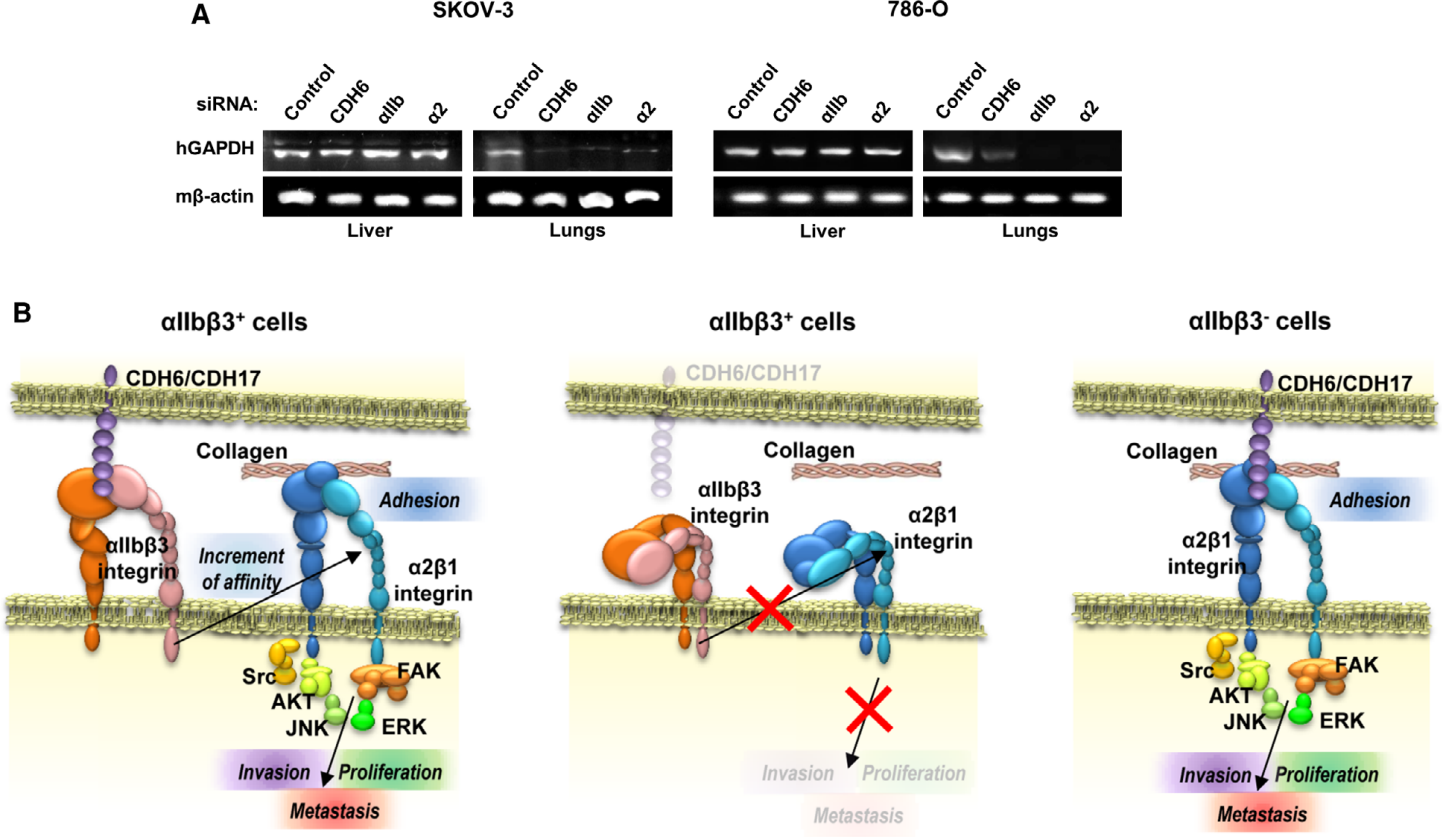
CDH6 and αIIb/α2 integrins are required for lung homing in ovarian and renal cancer. (A) RNA was isolated from liver or lungs of mice inoculated with SKOV‐3 cells previously silenced for the indicated genes and subjected to RT‐PCR assays to detect human GAPDH as surrogate of cell colonization. Murine β‐actin was used as loading control. (B) Model of integrin crosstalk. According to our results, the presence of CDH6 activates αIIbβ3 integrin, which induces the activation of α2β1 integrin. The activation of integrins promotes cell adhesion, invasion, and proliferation, leading to the metastatic dissemination of cancer cells.

Although peritoneal metastasis is the most common location for ovarian carcinomas, distant metastases of ovarian and renal cancers occur preferentially in lung. Thus, we investigated the effect of CDH6 silencing as well as integrin subunits αIIb and α2 on the homing capacity of ovarian and renal cancer cells in distant organs. Swiss nude mice were inoculated either intrasplenically or intravenously with siRNA‐silenced cells for liver and lung homing, respectively. Lungs and livers were collected 72 h after inoculation of transiently silenced SKOV‐3 and 786‐O cells, and RNA was extracted to amplify human GAPDH as a surrogate (Fig. 7A). In liver, GAPDH was detected to a similar extent as the three genes and control siRNA. In contrast, a barely or nondetectable band was observed in lungs from mice inoculated with cells siRNA‐silenced for the three genes. Therefore, the three proteins appear to be necessary for lung homing in ovarian and renal cancers. Taken together, our results reveal a key role for CDH6‐promoted αIIbβ3/α2β1 integrin crosstalk in adhesion, invasion, and lung metastasis in ovarian and renal carcinoma. The different models of integrin activation and crosstalk are summarized in Fig. 7B. In brief, the binding of CDH6, or CDH17, to αIIbβ3 integrin promotes α2β1‐mediated adhesion and invasion. However, the lack of αIIbβ3 activation inhibits α2β1 effects, except in those cells without αIIbβ3 expression.

## 4 Discussion

The molecular mechanisms underlying the progression and metastasis of ovarian and renal cancer are not fully understood. In this report, we have identified and characterized CDH6 and associated integrins as having a key role in ovarian and renal cancer progression. Our conclusions were based on the following observations: (a) a RGD‐dependent association of CDH6 and CDH17 with αIIbβ3 and α2β1 integrins, (b) this cadherin/integrin interaction promotes cell adhesion, migration, invasion, and proliferation through the activation of the SRC, FAK, AKT, and ERK pathway, (c) an integrin crosstalk in which αIIbβ3 regulates α2β1 activation for promoting adhesion and invasion, (d) the capacity of cadherin RGD‐specific mAbs and eptifibatide, an αIIbβ3 inhibitor, to block the invasive capacity of ovarian and renal cancer cells, and, finally, (e) the inhibition of lung homing in metastasis after cadherin/integrin silencing. In summary, we provide strong evidence of the potential therapeutic value of disrupting the interaction between CDH6 and the αIIbβ3/α2β1 integrins in ovarian and renal cancer metastases.

Two cell lines, CAKI‐1 and A‐2780, showed a quite different pattern of expression with respect to other renal and ovarian cancer cell lines. They showed no expression of CDH6 and very little amounts of α2 and αIIb subunits. Regarding CAKI‐1 cells, they form poorly differentiated G3 renal tumors that do not express α2 integrin [38], A2780 is the most commonly used model for a high‐grade serous ovarian carcinoma and is also considered an undifferentiated cell line [39]. Undifferentiated and poorly differentiated cancer cell lines do not express many epithelial cadherins. Therefore, our findings appear to be mainly applicable to differentiated metastases in ovarian and renal carcinomas. Two CDH6 isoforms were identified in ovarian cancer: the canonical full‐length (isoform 1) and isoform 2, which lacks the cytoplasmic tail. Little is known about the functions of CDH6 isoform 2 except its involvement in heterotypic interactions between osteoclasts and stromal cells, including morphological changes and tighter cell–cell associations [40]. We might think that isoform 2 could facilitate cell–cell homotypic interactions and maintain RGD‐mediated integrin activation without triggering the catenin pathways. Further studies are necessary to clarify the functional relevance of this isoform in cancer metastasis. It is also noteworthy that CDH17 was expressed in most of the tested cell lines. CDH17 has not been previously reported in Mullerian‐related tumors, although a clear overexpression of CDH17 can be observed in the mucinous subtype of ovarian cancer at the tissue level in the Human Protein Atlas database. There are few studies about the role of the cadherins in ovarian cancer; the loss of E‐cadherin and the increase of the mesenchymal phenotype were previously reported to promote ovarian cancer metastasis via α5β1 integrin [41]. Further studies will be necessary to investigate the correlation and potential interactions between E‐ and N‐cadherin with CDH6 and CDH17, to evaluate its impact on progression and metastasis.

Despite the high expression of α2β1 integrin in the plasma membrane of ovarian and kidney cancer cells, our results indicate a preferential binding of CDH6 to αIIbβ3 integrin whenever it is present. However, we observed that knocking down αIIbβ3 inhibits α2β1 integrin activity and, therefore, cell adhesion capacity. Although previously reported for leukocytes and platelets, this is (to the best of our knowledge) the first report of integrin crosstalk in solid cancers. Two different mechanisms of integrin crosstalk between αIIbβ3 and α2β1 have been reported in platelets [35, 42]: (a) αIIbβ3 activation is a key prerequisite for proper α2β1 activation via outside‐in signaling mechanism [35] and (b) a trans‐dominant inhibition, in which ligand binding to αIIbβ3 integrin inhibits α2β1‐mediated adhesion to collagen [33]. Our results suggest that ovarian and renal cancer cells follow the first mechanism, as αIIbβ3 silencing causes the inhibition of β1 activation and the loss of cell adhesion in SKOV‐3 and 786‐O cells. This situation could mimic the two‐step model proposed for platelet binding to collagen; the first step of Glycoprotein VI‐mediated activation of αIIbβ3 in platelets might be mimicked by CDH6 in cancer cells, which will result in the full activation of α2β1. Another effect of integrin crosstalk is the transactivation of growth factor receptors (e.g., EGFR or c‐Met) through cell adhesion and ligand‐independent phosphorylation of different growth factor receptors to increase cell proliferation [37]. In relation with this, a ligand‐independent activation of c‐Met by α5β1 integrin and fibronectin has been reported to regulate invasion and metastasis in ovarian cancer [43]. Indeed, c‐Met activates the SRC‐FAK pathway in a similar manner as that proposed by us for CDH6 and αIIbβ3 integrin. Therefore, we cannot discard a potential crosstalk between αIIbβ3 and α5β1 regulated by fibronectin, which would converge in the same pathway activation.

There is substantial evidence that platelets play a key role in ovarian cancer progression [44]. A platelet receptor, integrin αIIbβ3, is a well‐known metastasis‐associated molecule (see Ref. [45] for a review). Platelet αIIbβ3 is essential for pulmonary metastasis in various mouse models, as platelets may promote the arrest of tumor cells in the lung vasculature [45]. Moreover, although expression of αIIbβ3 integrin in cancer cells has been infrequently reported, it has been associated with lung metastasis [31, 46, 47]. Indeed, a pioneer report described the use of αIIbβ3 blocking mAbs for the inhibition of lung colonization in prostate cancer [31]. Our *in vivo* results also indicate a preferential activity of CDH6/αIIbβ3 interaction to enhance lung tropism and colonization. The duplication of CDH6, αIIbβ3, and α2β1 expression in cancer cells and platelets might facilitate a bidirectional interaction of cancer cells with platelets to facilitate the progression of the metastatic cascade. The possibility of blocking CDH6, αIIbβ3, or α2β1 integrins and their interactions opens new opportunities in metastasis treatment based on monoclonal antibodies or chemical inhibitors. So, mAbs 6.6.1 and 25.4.1 inhibited integrin activation induced by the CDH6 RGD motif, leading to the inhibition of pro‐metastatic activities and signaling pathways in both renal and ovarian cancer cells. Due to integrin crosstalk, mAbs appear to inhibit α2β1 activation either in a direct way or indirectly through αIIbβ3 to block the binding of α2β1 to collagen. Targeting the cadherin instead of the integrin would help to reduce the side effects of targeting integrins, derived from their important functions in homeostasis [48].

The therapeutic use of αIIbβ3 integrin inhibitors in cancer metastasis has been rarely investigated. A previous report showed the capacity of XV454, an oral antagonist of αIIbβ3 integrin, to inhibit lung metastasis in a mouse model of Lewis lung carcinoma [49]. This result was attributed to the inhibition of platelet aggregation and tumor cell‐induced thrombocytopenia. In our platelet‐free model, we observed a significant effect of the αIIbβ3 inhibitor eptifibatide on the adhesive and invasive capacities of SKOV‐3 and 786‐O cells. Eptifibatide was very effective at very low doses, which might prevent bleeding risks in patients. It has been described that eptifibatide antagonizes αIIbβ3 but also shows some potency on β1 integrins [50], likely due to the integrin crosstalk, which should potentiate its effect on blocking adhesion and invasion. Also, integrin crosstalk might explain why reagents specifically targeting αIIbβ_3_ likewise, inhibit (indirectly) the functions of α5β1 and α2β1 in the same cells [34]. In other words, αIIbβ3 inhibitors might block not only platelet aggregation but tumor‐platelet interactions and tumor cell adhesion/invasion in metastatic progression [51]. Therefore, the use of αIIbβ3 inhibitors appears to be a promising therapy for metastatic ovarian and renal cancers. The effects are likely mediated through both platelets and cancer cells.

## 5 Conclusions

In summary, we have demonstrated for the first time an integrin crosstalk between αIIbβ3 and α2β1 that regulates the metastatic progression in ovarian and renal cancer. This integrin crosstalk, promoted by CDH6, makes the targeting of the CDH6–αIIbβ3/α2β1 interaction a promising strategy for the development of novel therapeutic strategies. Overall, our study reveals an important mechanism of cancer progression mediated by CDH6 that affects the integrin signaling pathway activation in cancer cell adhesion and invasion. The antimetastatic capacity of clinically approved αIIbβ3‐specific inhibitors might facilitate a faster translation to cancer patients.

## Conflict of interest

JIC has stock ownership of Protein Alternatives SL. JR, CA, and JII are employees of Protein Alternatives SL. All other authors have no conflict of interest to declare.

## Author contributions

JIC and RAB designed the study. RAB, JR, AMR LP, MB, MJ, and CA carried out the experiments. RAB and JIC analyzed data. JII provided reagents, antibodies, and protocols; and RAB and JIC wrote the manuscript.

### Peer Review

The peer review history for this article is available at https://publons.com/publon/10.1002/1878‐0261.12947.

## Supporting information


**Fig. S1.** *In silico* analysis of CDH6 and αIIbβ3 integrin expression in renal and ovarian cancer.
**Fig. S2.** *In silico* prognosis studies of αIIb integrin subunit in renal and ovarian cancer.
**Fig. S3.** RGD cadherins and αIIbβ3 integrin modulate cell adhesion, migration, invasion and proliferation.
**Fig. S4.** CDH6 promotes cell adhesion, migration, invasion and proliferation in ovarian cancer cells.
**Fig. S5.** Crosstalk between RGD cadherins and α2β1 and αIIbβ3 integrins.

## Data Availability

The data that support the findings of this study are available in Figs 1, 2, 3, 4, 5, 6, 7 and the Supporting information of this article.
