# Supplementary material for: CDH6‐activated αIIbβ3 crosstalks with α2β1 to trigger cellular adhesion and invasion in metastatic ovarian and renal cancers
Source: Mol Oncol. 2021 May 2;15(7):1849–65. doi: 10.1002/1878-0261.12947 (PMC8253092; doi:10.1002/1878-0261.12947)
Supplement: Supplementary file 1 — Fig. S1. In silico analysis of CDH6 and αIIbβ3 integrin expression in renal and ovarian cancer. Fig. S2. In silico prognosis studies of αIIb integrin subunit in renal and ovarian cancer. Fig. S3. RGD cadherins and αIIbβ3 integrin modulate cell adhesion, migration, invasion and proliferation. Fig. S4. CDH6 promotes cell adhesion, migration, invasion and proliferation in ovarian cancer cells. Fig. S5. Crosstalk between RGD cadherins and α2β1 and αIIbβ3 integrins. [file MOL2-15-1849-s001.pdf]

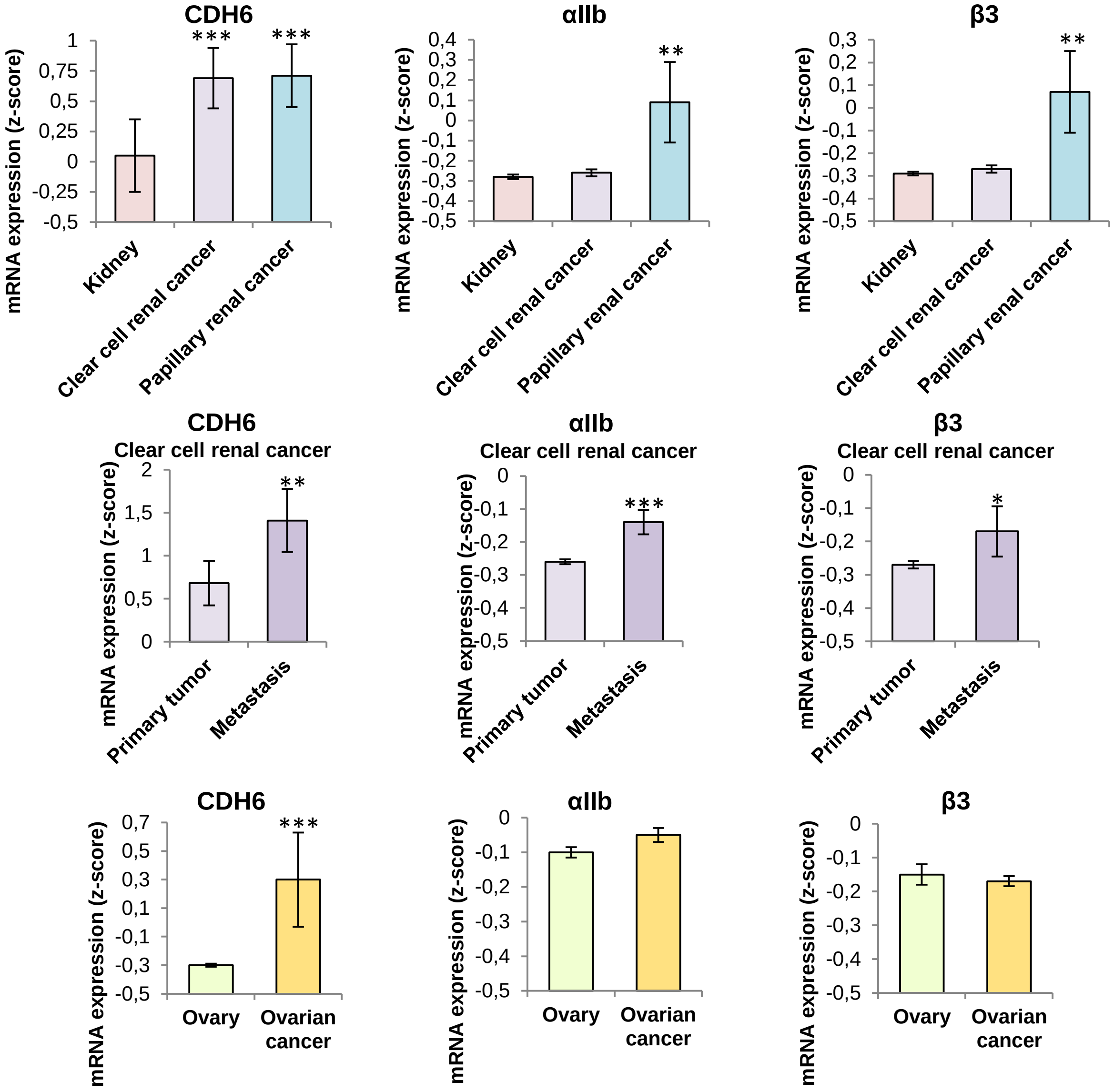

Figure S1. In silico analysis of CDH6 and αIIbβ3 integrin expression in renal and ovarian cancer. For renal cancer, expression of the studied genes was analyzed with Oncomine platform using TCGA Renal 2 database (with 1071 tumor samples and 441 healthy kidney samples). For clear cell renal carcinoma, differences in the expression of the studied genes between primary tumors and metastasis were tested using the public database GSE22541, which contains 68 samples (44 metastasis and 24 primary clear-cell renal carcinomas). For ovarian cancer, public database GSE26712 (with 195 tumor samples and 10 healthy ovarian samples) were used for the analysis. The values in mRNA expression levels were normalized by calculating the z-scores, and the differences were assessed by Student's t test (for two sample types) or one-way ANOVA (for three sample types). Expression of the indicated genes was significantly enhanced in tumor samples compared to healthy tissues, or metastatic tumors compared to primary tumors (*, $p < 0.05$; **, $p < 0.01$; ***, $p < 0.001$) according to ANOVA tests. Error bars indicate standard error of the mean.

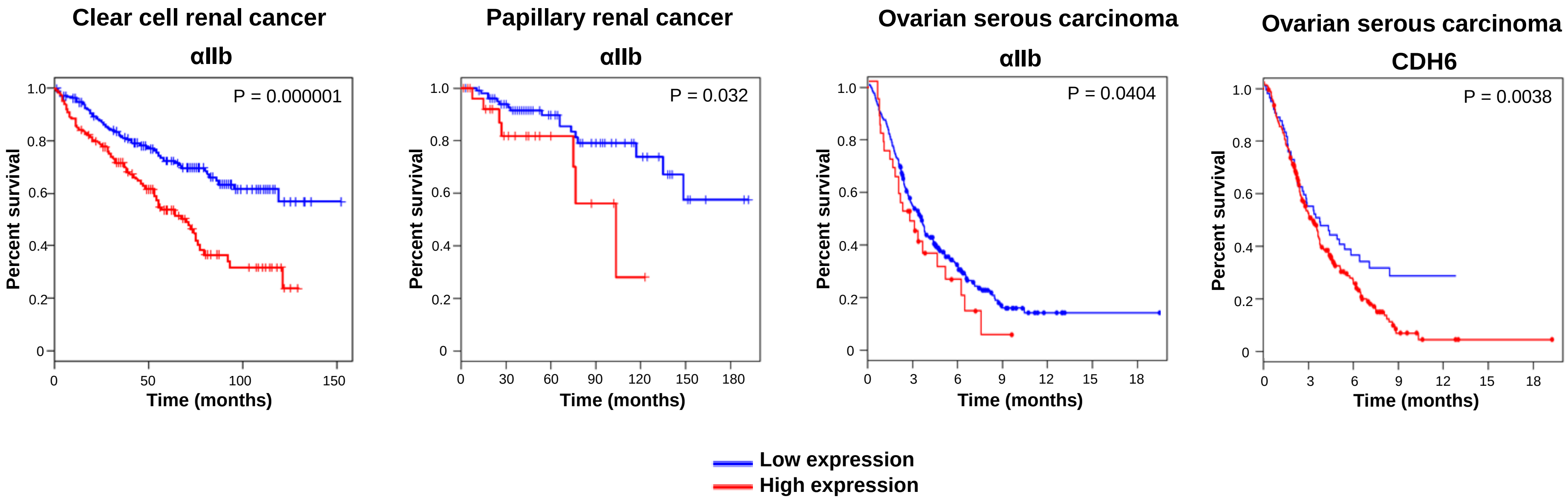

Figure S2. In silico prognosis studies. Independent external cohorts of patients were used for in silico analysis of patient survival. Public databases GSE26193 and GSE26712, which contain 107 and 195 tumor samples, respectively, from chemotherapy treated ovarian cancer patients were used for the analysis. Data about expression levels of mRNA were analyzed by calculating the z-score for each studied gene and sample, and normalized before joining both databases. Populations were divided into high and low expression by best-cut off method. For renal cancer, GEPIA web tool was employed for analysis of TCGA databases, which contain 523 and 310 tumor samples for clear cell and papillary renal cancer, respectively. The prognostic value of each gene was visualized using Kaplan-Meier survival curves and assessed by Log-rank test. P value of the test is indicated inside each panel.

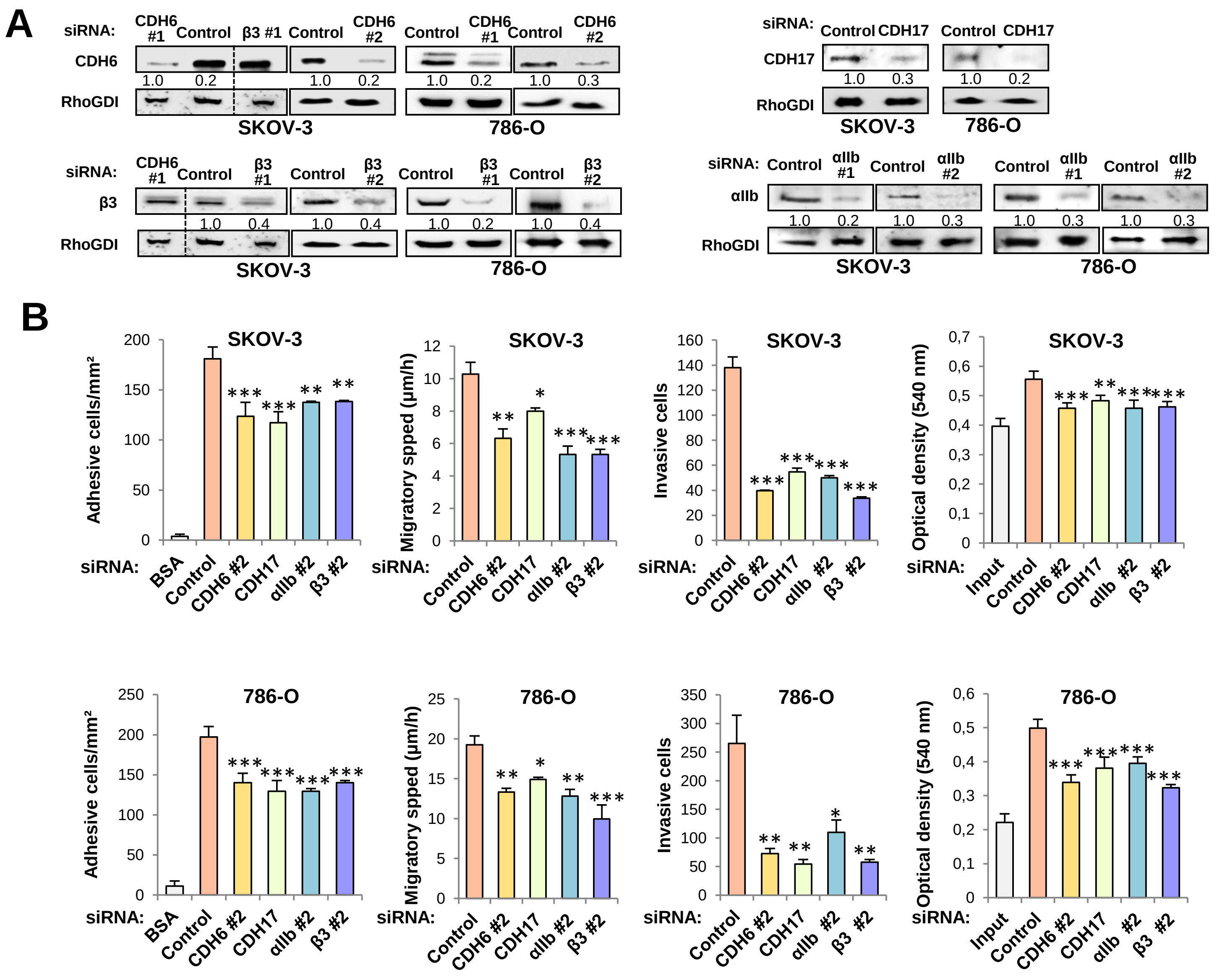

Figure S3. RGD cadherins and αIIbβ3 integrin modulate cell adhesion, migration, invasion and proliferation. (A) The indicated cell lines were transfected with siRNAs against CDH6, CDH17, αIIb and β3 integrins or a control siRNA. Silencing in the expression of these genes was assessed by Western blot. RhoGDI was used as loading control. (B) The same transfectants were subjected to adhesion to Matrigel, wound healing, invasion through Matrigel and MTT assays. Cell adhesion, migratory speed, number of invasive cells and optical density were significantly inhibited by the silencing of the indicated genes (*, $p < 0.05$; **, $p < 0.01$; ***, $p < 0.001$) according to ANOVA tests. Error bars indicate standard deviation.

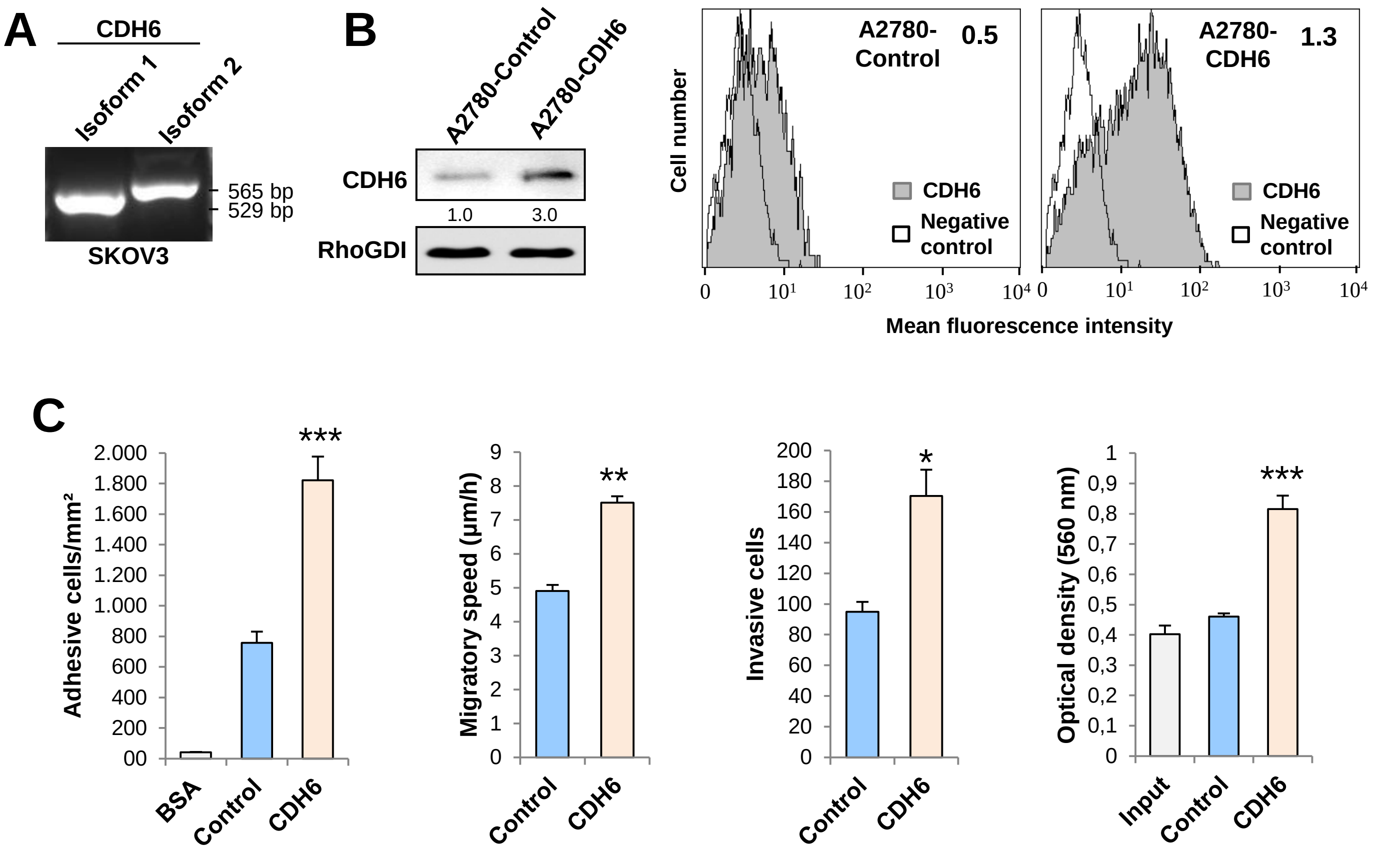

Figure S4. CDH6 promotes cell adhesion, migration, invasion and proliferation in ovarian cancer cells. (A) RT-PCR analysis to assess the expression of CDH6 isoforms 1 (long) and 2 (short) in SKOV3 cells. (B) A-2780 cells were infected with lentiviral vectors encoding for CDH6 or empty vectors (control). After infection, cells were subjected to Western blot (left) or flow cytometry analyses to assess the expression of CDH6. (C) The same stable transfectants were subjected to cell adhesion, wound healing, cell invasion or MTT assays. CDH6 expression caused a significant increase in cell adhesion/migration/invasion/proliferation (*, $p < 0.05$; **, $p < 0.01$; ***, $p < 0.001$) according to ANOVA tests. Error bars indicate standard deviation.

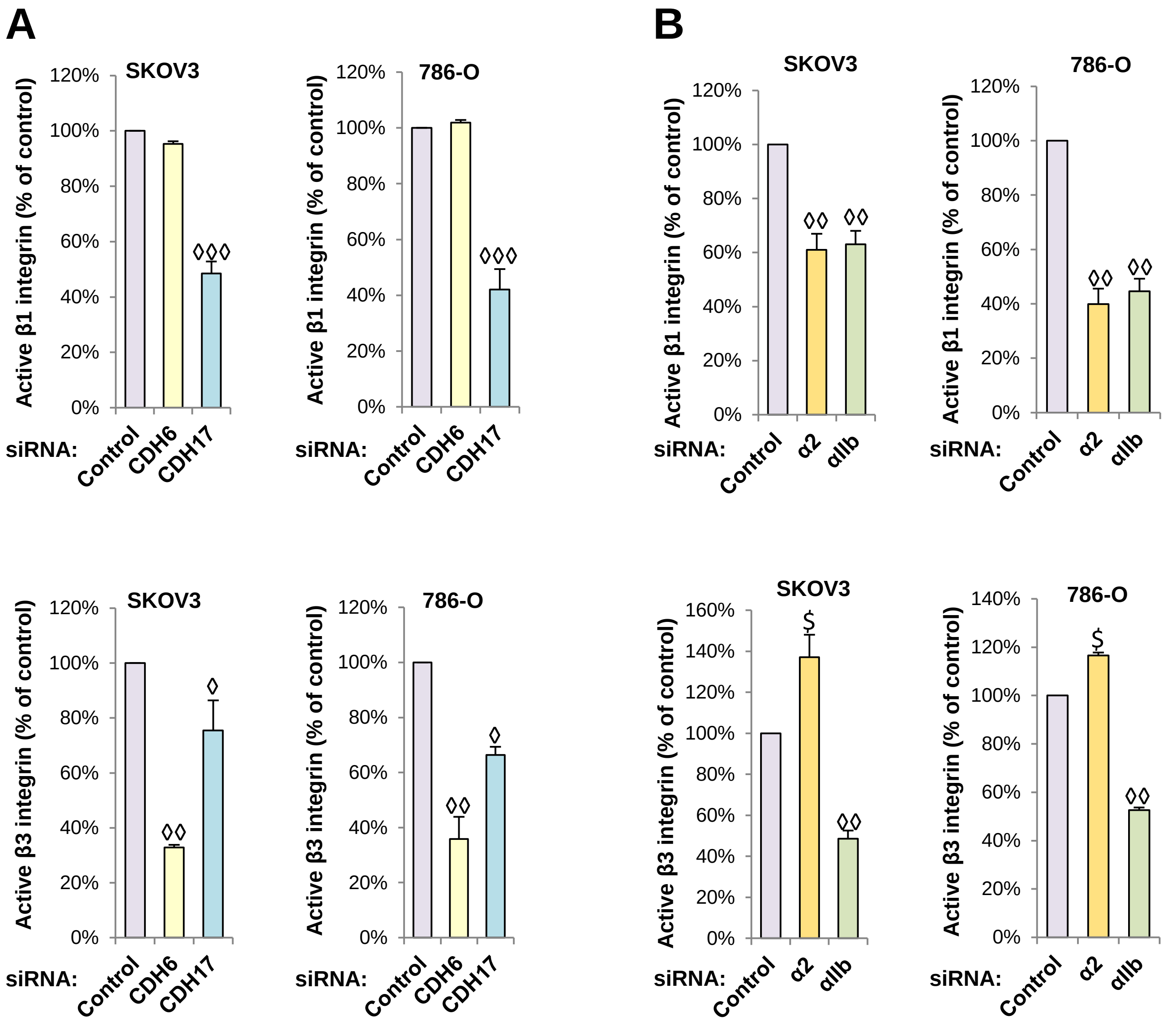

Figure S5. Crosstalk between RGD cadherins and α2β1 and αIIbβ3 integrins. (A) The indicated cell lines were transfected with siRNAs against CDH6, CDH17, or a control siRNA. Transfectants were subjected to flow cytometry analyses to detect β1 and β3 integrins in high affinity conformation. (B)) The indicated cell lines were transfected with siRNAs against α2, αIIb integrins, or a control siRNA. Transfectants were subjected to flow cytometry analyses to detect β1 and β3 integrins in high affinity conformation. Silencing of cadherins or integrins significantly inhibited integrin activation (◊, $p < 0.05$; ◊◊, $p < 0.01$; ◊◊◊, $p < 0.001$), whereas silencing of α2 integrin significantly induced the activation of β3 integrin according to ANOVA tests. Error bars indicate standard deviation.
